# The Story of the Insane from Year to Year

**Published:** 1904-09-17

**Authors:** 


### THE STORY OF THE INSANE FROM YEAR TO YEAR.

### Sixteenth Annual Series.

### LONDON COUNTY ASYLUM, EPSOM.

There were 749 patients in residence on the first of January, and on the 31st December the number had fallen to 695. The first admissions were 189, the readmissions 6, the recoveries 41, the discharged relieved 11, the discharged not improved 129, and the deaths 68. The average number resident during the year was 685, and the total number under treatment was 944. Calculated on the admissions the recoveries stand at 26·45 per cent.; and the deaths calculated on the average number resident at 9·93 per cent. Of the year's deaths 27 were caused by cerebral and spinal diseases, 12 by consumption, 4 by pneumonia and 7 by colitis. Various moral causes account for the insanity in 53 of the year's admissions; and among the physical causes hereditary influence heads the list with 40, intemperance in drink comes next with 31, and in 52 no cause was ascertained. This asylum is intended for female patients only, and provision is made for 117 private patients. The latter supplies a real want in the London districts; and the charges ought to be only a trifle higher than the pauper maintenance rate plus a sum for lodging. The committee have also wisely decided to erect a separate block for the accommodation of 60 male patients, and before this notice is in type the block will have been completed and occupied. Dr. Donaldson does not say much about the 7 deaths from colitis, merely remarking that the disease appeared from time to time, and that the patients attacked were "usually old and broken down dements." The committee record their "appreciation of the efficient manner in which Dr. Donaldson and the medical and general staff have discharged their duties." The weekly rate of maintenance was nearly 11s. 10d. a head.

### LONDON COUNTY ASYLUM AT HORTON.

THIS asylum was opened on the 3rd March, 1902, and on the last day of December it contained 1,779 patients. There were 2,015 first admissions, 4 readmissions, 88 discharged recovered; 44 were discharged relieved, and 108 died. The average number resident was 958, and the total number under treatment was 2,119. Calculated on the admissions the recoveries were 13 43 per cent. and calculated on the average number resident the deaths were 11·27 per cent.; but as the asylum had not been opened a year such percentages are of no value to compare with those of other institutions. Of the deaths 32 were caused by cerebral and spinal diseases, 14 by consumption, 9 by pneumonia, and 3 by colitis. Of the admissions 231 owed their insanity to such moral causes as domestic trouble, adverse circumstances, and mental anxiety. Among the physical causes hereditary influence is credited in 211 cases, and intemperance in drink in 201. Dr. Bryan's duties during the year must have been onerous in the extreme; and we are not surprised to hear that his chief difficulties consisted in procuring suitable people for his staff. A medical superintendent, under such circumstances, has to be satisfied with second-rate material, even must he draw upon that "floating population of nurses and attendants who, knowing the condition of affairs, seem to delight in moving from one asylum to another." When his staff gets more settled, Dr. Byran will be well advised to steer entirely clear of anyone who has been employed in any other asylum unless the circumstances are very, very exceptional. As already said the deaths included three from colitis; but we are not enlightened as to the real or supposed cause of the outbreak. Here is a new asylum, with, presumably, the sanitary arrangements as good as they could possibly be, and yet this troublesome disease makes its ap[illegible]arance within a month or two of its opening, The weekly [illegible]te of maintenance was 11s. 7d. per head.

### LONDON COUNTY ASYLUM AT HANWELL.

ON the first of January this asylum contained 2,545 [illegible]tients, and on the 31st of December the number had risen [illegible] 2,556. There were 411 first admissions, 86 readmissions, [illegible] discharged recovered, 20 discharged relieved, and 222 [illegible]aths. The total number under treatment during the year [illegible]s 3,042, and the average daily number resident was 2,544. [illegible]cluding transfers the recoveries stand at 37·45 per cent. [illegible] the admissions, and the deaths at 8·73 per cent. of the [illegible]erage number resident. Of the year's deaths 111 were [illegible]used by cerebral and spinal diseases, 26 by consumption, [illegible] by other lung diseases, and 8 by dysentery. Of the year's [illegible]missions various moral causes are put down as pro[illegible]cing the insanity in 53 instances, and among the physical [illegible]uses heredity is credited with 92, previous attacks with 96, [illegible]temperance in drink with 70, and in 53 the disease was of [illegible]known origin. As regards the 8 deaths from dysentery, [illegible]r. Alexander points out that the total number of cases, [illegible] compared with the previous year, has fallen from 64 to [illegible], and he hopes that this is merely an earnest of a still further decrease. We hope so too; but this asylum dysentery has a curious habit of disappearing and reappearing without apparent cause. In the meantime Dr. Alexander is doing everything to stamp out the disease that experience can suggest. Superannuation allowances were granted to members of the staff in 10 cases, but the amounts given are not stated. The committee record their "appreciation of the care and kindness bestowed on the patients by Dr. Alexander." The weekly rate of maintenance was a fraction under 11s. per head.

### WEST RIDING ASYLUM, WAKEFIELD.

AT the beginning of the year this asylum contained 1,658, and at the end of the year the number had risen to 1,805, the full accommodation being for 1,863. If we take the low estimate of 2 per cent. of the number resident as the annual increment, the asylum will again be full within two years. Last year the first admissions were 515, the readmissions 129, the recoveries 245, and the deaths 183. The average number resident was 1,741, and the total number under treatment during the year was 2,302. The recoveries were 38 per cent. of the admissions, but excluding the transfers this percentage would rise to 44 88. The deaths were 10·51 per cent. of the average number resident. Of the deaths, 67 were caused by cerebral and spinal diseases, general paralysis alone accounting for 43 of this number; consumption caused death in 21 cases, other lung diseases in 21, colitis in 7, and enteric fever in 1. Such moral causes as domestic trouble, adverse circumstances, were supposed to have caused the outbreak of insanity in 127 of the admissions, and of the physical causes hereditary tendency heads the list with 268, and intemperance in drink follows with 150. As already stated, 7 of the deaths were due to colitis or asylum dysentery; and this affords a percentage of 3 8 on the total deaths, and contrasts favourably with the percentages of the two previous years, which were 6 per cent. and 4 per cent. respectively; but with all his care and precautions Dr. Lewis has not succeeded in banishing this scourge from his death tables. When referring to the deaths from consumption, Dr. Lewis expressed the opinion that it would be better and more economical to erect a cheap structure at each of the West Riding asylums rather than a more pretentious building common to them all; and he is almost certainly right in this opinion. There are seven medical men on the staff, and there would seem to be much medical activity throughout the asylum. The committee of visitors state that "Dr. Bevan Lewis' excellent management continues to deserve the highest approbation." The weekly cost of maintenance is 11s. 2d. per head.

